# Fear of Uncertainty Makes You More Anxious? Effect of Intolerance of Uncertainty on College Students’ Social Anxiety: A Moderated Mediation Model

**DOI:** 10.3389/fpsyg.2020.565107

**Published:** 2020-09-08

**Authors:** Jie Li, Ying Xia, Xinying Cheng, Shijia Li

**Affiliations:** ^1^School of Psychology, Jiangxi Normal University, Nanchang, China; ^2^Center of Mental Health Education and Research, Jiangxi Normal University, Nanchang, China

**Keywords:** social anxiety, intolerance of uncertainty, rumination, pessimistic explanatory style, moderated mediation model, college students

## Abstract

**Purpose:**

This study investigated the relationships among intolerance of uncertainty (IU), social anxiety (SA), rumination, and pessimistic explanatory style (PES) in a sample of college students.

**Methods:**

Questionnaires were completed by 533 college students.

**Results:**

Rumination partially mediated the relationship between IU and SA, and PES plays an important role in moderating the direct path (IU→SA) and the first part of the mediation process. When the PES is low, IU predicts SA less strongly but is related to rumination. Conversely, IU in individuals with a high PES predicts SA more directly.

**Conclusion:**

Rumination plays a mediating role between IU and SA, and the PES moderates the direct path and the first stage of the mediation model.

## Introduction

Social anxiety (SA) refers to individuals’ strong, persistent, and irrational fear of being exposed to social situations (Hyett and McEvoy, 2018) and is one of the most common forms of anxiety. Chinese researchers emphasized college students are the main group affected by SA in China (Guo, 2000), because this age group has the highest interpersonal sensitivity (Peng et al., 2003). The local studies explored the trend of the social anxiety level of Chinese college students and found that compared with 1998, the score of social anxiety in 2015 increased by 0.27 standard deviations (Shi and Xin, 2018). In addition, there about 16% of college students report that they have serious social anxiety, which affects their basic life (Xu, 2010). SA can have many adverse effects on college students such as reducing their quality of life, subjective well-being, friendship quality, and academic performance (Jia et al., 2019; Zhang et al., 2019). According to the etiological explanation model of anxiety (Taylor and Wald, 2003), IU is a specific influencing factor of anxiety. Another study described a significant positive correlation between IU and SA. The authors reported that IU contributes 4% of the explainable variance after controlling the fear of negative evaluation, anxiety, sensitivity, and neuroticism (Boelen and Reijntjes, 2009). As a personality factor, IU can reflect the tendency of individuals to produce negative beliefs when facing uncertainty (Dugas and Robichaud, 2007). Moreover, IU is the main antecedent variable of SA, and it greatly affects our daily life. IU can explain why some people can persist through, actively respond, and adapt to uncertain situations, while some others show excessive worry, anxiety, depression, and even difficultly normally processing information in social circumstances (Flores et al., 2018). Based on this, we attempted to explore how IU influences SA. The internal mechanism of the relationship between IU and SA is also worth further discussion.

The SA cognitive model proposed by Clark and Wells (1995) Clark and Wells suggests that automated negative thinking plays a crucial role in SA. Individuals who cannot tolerate uncertainty are more likely to respond to stress in social situations with repetitive thinking such as worry or rumination (Morgan and Banerjee, 2008; Yook et al., 2010). Rumination is defined as “persistent” thinking about their own experience, the emotional causes, and various adverse consequences of their negative coping style (Nolen-Hoeksema et al., 2008; Pont et al., 2018). Rumination has a significant positive effect on SA (Sergiu and Aurora, 2015) and is recognized as an important factor to trigger, maintain, and accelerate SA based on the theory of reaction style (Fang and Sun, 2018). When we focus on the role of “rumination” in the relationship between IU and SA, we find that it is conceptualized as an intermediary between cognitive risk factors (e.g., IU) and negative psychological outcomes (e.g., depression), which means individuals with high IU are likely to consistently ruminate to cope with their negative emotions (Spasojeviæ and Alloy, 2001). Thus, we reasonably speculate that the passive coping response of rumination is the underlying link between IU and SA. Based on the above theories and literature support for the relationship among IU, rumination, and SA, we propose Hypothesis 1: Rumination plays a mediating role between IU and SA.

Socially anxious (SA) individuals interpret ambiguous social events negatively (Amir et al., 2005), which shows the importance of explanatory style in the interpretation of uncertain events in SA groups. Therefore, it leads us to explore whether the relationship between IU and SA is also susceptible to explanatory style. From the perspective of information processing, a high pessimistic explanatory style (PES) strengthens the processing bias of uncertain information, which makes individuals more likely to experience anxiety (Kaur, 2017). This prompted us to include PES as a moderating variable when exploring the relationship between IU and SA. PES is generally considered a way of interpreting the cause of negative life events in an internal, stable, and universal way. Individuals with a PES pay more attention to negative information and have negative predictive effects on mental health such as depression and anxiety (Cheng and Furnham, 2001). Individuals with lower PES are more likely to interpret negative information using external, unstable, and special interpretations and are less susceptible to cognitive interference from negative information (Abramson et al., 1989). It alleviates the negative impact of uncertain information on individual cognition and emotional outcomes. Thus, we believe that PES may play a moderating role in the relationship between IU and SA. According to the theory of self-regulating executive function (Matthews and Wells, 2000), PES is an important factor affecting rumination. However, how the moderating role of PES will affect a mediation model including IU, SA, and rumination is unknown. It is universally believed that people with a PES are habitually negative thinkers, which makes it difficult for them to withdraw from internal self-regulation, and they become trapped in rumination. Whether this reinforcing effect can be verified in the relationship between IU and rumination remains to be explored. Given the special impact of PES on rumination, we predict that PES plays a moderating role between IU and rumination. When the first or second half of the mediation path is moderated, the mediation effect can also be moderated (Wen and Ye, 2014). Based on this, we propose Hypothesis 2: PES moderates the direct prediction effect of IU on SA, and the mediating effect of rumination is moderated by the PES.

**FIGURE 1:**
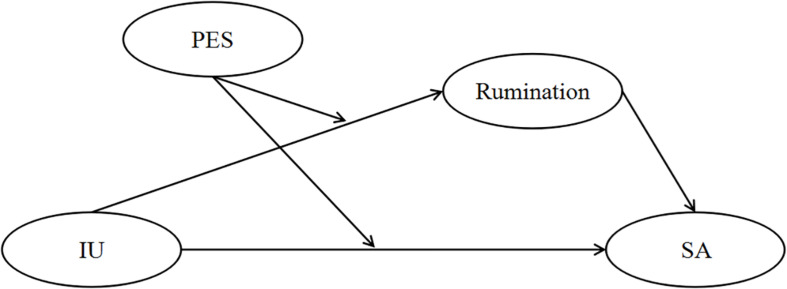
The mediating role of rumination and moderating role of PES. IU, intolerance of uncertainty; PES, pessimistic explanatory style; SA, social anxiety.

In summary, the present work is based on the anxiety etiology interpretation model and the SA cognitive model proposed by Clark and Wells (1995) and intends to integrate the theory of reaction style and the theory of self-regulating executive function. The goals of this study are to explore the relationship between IU and SA and its internal mechanism, focusing on the mediating role of rumination and the moderating role of PES. Clarifying the relationship between IU and SA will provide theoretical support for clinical treatment of SA. The model diagram of our moderated mediation function hypothesis is shown in Figure 1.

## Materials and Methods

### Participants and Procedure

This study used a cluster sampling method. We conducted on-site surveys of undergraduates in three different grades. Participants were recruited via public courses from our university. All participants signed an informed consent form before completing the questionnaires, and questionnaire instructions were explained by professionally trained personnel. Participants were required to respond to all questionnaire items honestly according to their experience in daily life. After confirming that participants understood the instructions, questionnaires were completed independently and collected on the spot. A total of 570 questionnaires were distributed, and 533 valid questionnaires were recovered (93.50% recovery rate). Among the valid questionnaires, participants included 233 males (43.71%) and 300 females (56.29%); 430 freshmen (80.67%), 75 sophomores (14.07%), 27 juniors (5.07%); 166 only children (31.14%), and 367 non-only children (68.86%). The respondent age ranged from 18 to 23 years, with a mean of 19.49 (SD = 1.07). This study was reviewed and approved by the Moral & Ethics Committee of School of Psychology, Jiangxi Normal University (Nanchang, China).

### Measures

#### Intolerance of Uncertainty

The Intolerance of Uncertainty Scale (IUS) was compiled by Freeston et al. (1994), and the English version was revised by Buhr and Dugas (2002). This study used the 11-item Chinese version revised by Li et al. (2015). A five-point Likert-type scale was used for scoring, with 1 indicated “complete non-conformity” and 5 means “completely consistent.” A higher total score corresponded to higher IU. The internal consistency coefficient of the IUS in this study was 0.85, indicating good internal consistency.

#### Rumination

The 22-item Ruminative Responses Scale (RRS) was compiled by Nolen-Hoeksema and Morrow (1991) and revised by Han and Yang (2009). A four-point scoring method was used, with 1 means “occasionally” and 4 means “continuously.” A higher total score indicated more severe rumination. The internal consistency coefficient of the RRS in this study was 0.90, indicating excellent internal consistency.

#### Explanatory Style

The Attributional Style Questionnaire (ASQ) compiled by Peterson et al. (1982) and revised by Wen (2007) was based on college students. This study used the Negative Interpretation Style subscale. The questionnaire contains 6 items and a 7-point scoring method was used. For example: “You’re sick. The reason why you are sick: 1 = Because of external factors, 7 = Because of yourself. 1 = No longer exists, 7 = Always exists. 1 = Only affects such events, 7 = Affects all.” The questionnaire includes three independent dimensions and one comprehensive dimension. The scores of the three independent dimensions are: the average score of internal evaluation (IN), stability evaluation (SN), general evaluation (GN) of six negative events. The score of the comprehensive dimension (CN) is, respectively, add the scores of negative events in the three dimensions and divide by the number of negative events. A higher total score indicated higher pessimistic explanatory. The internal consistency coefficient of the ASQ in this study was 0.79, indicating good internal consistency.

#### Social Anxiety

The 28-item self-rating social anxiety scale compiled by Yang (2003) was based on college students. The questionnaire uses a five-point scoring method, ranging from “0 = completely inconsistent” to “4 = completely consistent.” A higher score indicated a higher degree of SA. The internal consistency coefficient of the SA scale in this study was 0.94, indicating excellent internal consistency.

### Data Analysis

All questionnaires were scored positively. The prior procedural control process of test and common variance analysis were applied to the four questionnaires through the Harman’s single-factor test. Using SPSS 21.0 (IBM Corp., Armonk, NY, United States) statistical software, the correlations between variables were tested using Pearson correlations after descriptive statistics had been computed. Hypotheses 1 and 2 were tested using moderated mediation analyses via the SPSS macro program PROCESS (written by F. Andrew and edited by Hayes, 2013). Model 4 was used to test Hypotheses 1, and Model 8 was used to test Hypotheses 2. To determine how PES moderates the relationship between IU, rumination, and SA, a simple slope test was used. The interaction diagram based on psychological detachment was adopted (one standard deviation above the mean and one standard deviation below the mean).

## Results

### Control and Verification of Common Method Variance

The Harman single-factor test was used to test common method deviation (Podsakoff et al., 2003). The results revealed 28 eigenvalues >1 without rotation, and the mutation rate interpretation of the first factor was 14.27%, which was less than the critical value of 40%, indicating that there was no obvious deviation of the common method in this study.

### Descriptive Statistics

The correlation matrix for each variable is shown in Table 1. Correlation analysis showed that IU was significantly positively correlated with SA, rumination and PES. PES was significantly positively correlated with rumination. Rumination was significantly positively correlated with SA.

**TABLE 1:** Means, standard deviations, and correlation coefficients (*n* = 533).

	*M*	*SD*	1	2	3	4	5	6
(1) Gender^a^	0.57	0.50	1					
(2) Age	19.49	1.07	0.05	1				
(3) Intolerance of uncertainty	31.19	7.18	0.06	–0.05	1			
(4) Pessimistic explanatory style	4.55	0.60	0.02	–0.02	0.12**	1		
(5) Rumination	45.11	10.20	0.06	−0.16***	0.34***	0.21***	1	
(6) Social anxiety	52.58	10.63	-0.05	–0.07	0.39***	0.15**	0.49***	1

*p < 0.05; **p < 0.01; ***p < 0.001. M, mean; SD, standard deviation.

### The Relationship Between IU and SA: A Moderated Mediation Model

The mediation effect was tested before assessing moderation effects (Wen and Ye, 2014). Therefore, Model 4 (a simple mediation model) in the SPSS expansion macro prepared by Hayes (2012) was first used firstly to test the mediation effect of rumination on the relationship between IU and SA. IU was a significant predictor of SA (β = 0.57, *t* = 9.67, *p* < 0.001, [CI] = [0.46,0.69]), and IU can remained predicted SA when both IU and ruination were entered into the regression equation (β = 0.37, *t* = 6.50, *p* < 0.001, CI = [0.26,0.49]). IU had a significant positive predictive effect on rumination (β = 0.48, *t* = 8.23, *p* < 0.001, CI = [0.36,0.59]), and rumination was a significant predictor of SA (β = 0.42, *t* = 10.30, *p* < 0.001, CI = [0.34,0.50]). Therefore, rumination plays a partial mediating role in the relationship between IU and SA. The direct (0.38) and mediated (0.20) prediction effects accounted for 65.52 and 34.48% of the overall effect, respectively. Thus, Hypothesis 1 was supported.

In the second step, we employed Model 8 in the SPSS extension macro (Model 8 moderates the direct path and the first stage of the mediation model, which is consistent with the hypothetical model in this study), and the moderated mediation model was tested. As shown in Table 2, after inputting PES into the model, the interaction between IU and PES was a significant predictor of rumination (IU × PES: β = −0.25, *t* = −2.81, *p* < 0.01), and the interaction was also a significant predictor of SA (IU × PES: β = 0.22, *t* = 2.56, *p* < 0.05), indicating that PES moderated the relationship between IU and SA (Model 1) and the relationship between IU and rumination (Model 2).

**TABLE 2:** Moderated mediation effect analysis of the relationship between IU and SA.

	Model 1 (criterion: SA)				Model 2 (criterion: Rumination) 2			

	*B*	*SE*	β	95% CI	*B*	*SE*	β	95% CI
IU	0.36	0.06	0.36***	[0.25, 0.47]	0.45	0.06	0.46***	[0.34, 0.57]
PES	0.59	0.66	0.59	[-0.70, 1.89]	2.91	0.69	2.91***	[1.57, 4.26]
IU × PES	0.22	0.08	0.22*	[0.05, 0.38]	-0.25	0.09	-0.25**	[-0.43, -0.08]
Rumination	0.42	0.04	0.42***	[0.34, 0.50]				
*R*^2^	0.30				0.17			
*F*	57.00***				21.67***			

*p < 0.05; **p < 0.01; ***p < 0.001. B, regression coefficient; CI, confidence interval; IU, intolerance of uncertainty; PES, pessimistic explanatory style; SA, social anxiety; SE, standard error.

**FIGURE 2:**
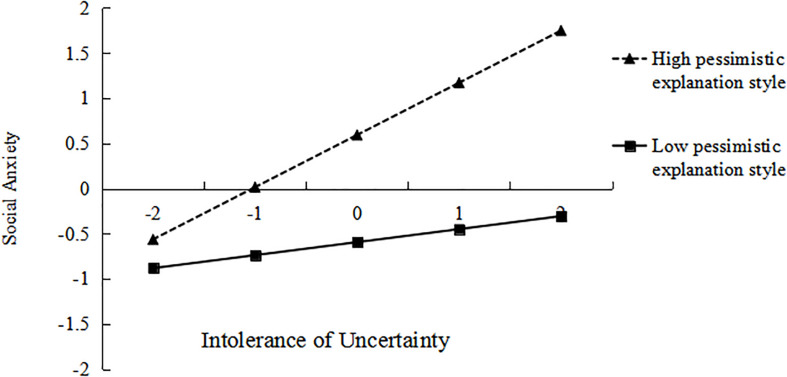
Moderating role of pessimistic explanation style on the relationship between intolerance of uncertainty and social anxiety.

To understand how the moderator works, simple slope analysis was carried out as shown in Figure 2. The relation between IU and SA was more positive under high level of PES (*M* + *1SD*; β_simple_ = 0.14, *t* = 2.59, *p* < 0.01) than that under low level of PES (*M*−*1SD*; β_simple_ = 0.58, *t* = 10.69, *p* < 0.001). Table 3 shows the direct and indirect effects of IU on SA in groups with low and high level of PES. Hence, the results indicated that increasing the level of PES can strengthens the association between IU and SA.

**TABLE 3:** The direct and indirect effects of IU on SA for different levels of pessimistic explanation style.

	PES	*B*	*SE* 2	95% bootstrap CI 4 95% bootstrap CI 6 4 6
Direct predictive effect	-0.6(*M*-*1SD*)	0.23	0.08	[0.07, 0.39]
	0.00(*M*)	0.36	0.06	[0.25, 0.47]
	0.60(*M* + *1SD*)	0.49	0.07	[0.35, 0.64]
Mediation effect of rumination	-0.6(*M*-*1SD*)	0.25	0.04	[0.17, 0.34]
	0.00(*M*)	0.19	0.04	[0.12, 0.27]
	0.60(*M* + *1SD*)	0.13	0.05	[0.04, 0.24]

*B, regression coefficient; CI, confidence interval; M, mean; PES, pessimistic explanatory style; SD, standard deviation; SE, standard error.*

**FIGURE 3:**
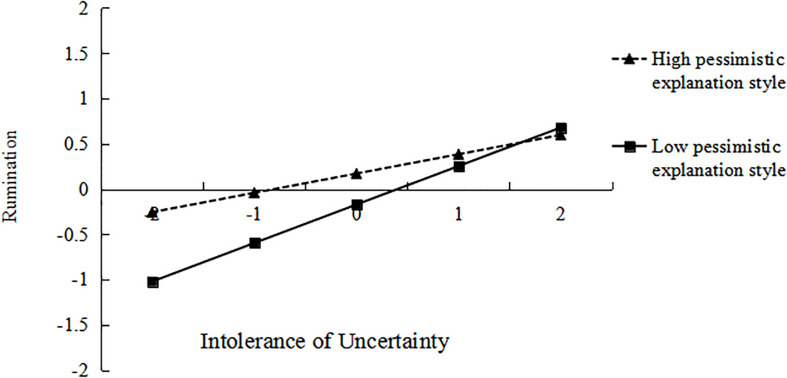
Moderating role of pessimistic explanation style on the relationship between intolerance of uncertainty and rumination.

As shown in Figure 3, the relation between IU and rumination was more positive under low level of PES (*M*−*1SD*; β_simple_ = 0.43, *t* = 7.62, *p* < 0.001) than that under high level of PES (*M* + *1SD*; β_simple_ = 0.21, *t* = 3.92, *p* < 0.001). Hence, the results indicated that increasing the level of PES can weakens the association between IU and rumination. In summary, the mediation effects of rumination were increased and decreased for low and high level of PES, respectively. It means with the levels of PES increasing, the mediation effect between IU and SA showed a downward trend, IU was less likely to induce SA by increasing rumination.

## Discussion

Based on the cognitive behavior model of SA proposed by Clark and Wells (1995) and the theory of self-regulating executive function proposed by Matthews and Wells (2000), this study examined the mediating effect of rumination between IU and SA, and the moderating effect of PES in this relationship.

The results showed that IU positively predicted individuals’ SA levels. The further tests on the mediating effect of rumination showed that rumination played a partial mediating role between IU and SA. This result support the hypothesis 1 and consistent with previous similar findings (Liao and Wei, 2011). It is proved that IU can directly predict SA or indirectly predict SA through rumination. Individuals with high IU levels are more likely to make more threatening interpretations of fuzzy information than individuals with low IU levels (Dugas et al., 2005), and those who can’t bear the uncertainty often think social scene is threatening and out of control within their own ability (Li et al., 2014), so the high level of IU tend to form a high level of SA. Also, IU is more likely to cause aberrant information processing, the repeated thinking of rumination exacerbates the depolarization of deviating information (Andersen and Limpert, 2001), and solidifies the SA result (Teivaanmäki et al., 2018). All these results show that rumination serves as a bridge between IU and SA (Werner et al., 2011). This conclusion is in line with the cognitive behavior model of SA (Rapee and Heimberg, 1997) and support the prediction of rumination can act as a mediator between IU and negative psychological outcomes by Spasojeviæ and Alloy (2001).

The results of this study indicate that the PES can regulate the direct path and the first stage of the mediation model (IU → rumination → SA). PES moderated the relationship between IU and SA, indicating that the direct predict of IU on SA is more significant for high PES individuals than for low PES individuals. This suggests that there are individual differences in the intrinsic mechanism of SA. It also indicates that PES is a cognitive factor leading to SA in individuals, which is consistent with a previous study (Cheng and Furnham, 2001), which is consistent with previous studies (Morrison and Heimberg, 2013). IU can induce individual to negatively explain the vague information in social situations and cause anxiety. Individuals with high level of PES more likely to “store” the pessimistic explanation of this threat in an internal, stable, and universal way, thus reinforcing their SA. This extrapolation stems from the explanation of high PES by the despair theory (Abramson et al., 2002). Therefore, a high PES can enhance the cognitive damage of negative information caused by IU, in turn enhancing the association between IU and SA.

In addition, this study found that PES also acted as a moderator in the first part of the mediation process (IU → rumination). Higher IU is more likely to be linked to higher rumination in individuals with low PES, resulting in the emergence of SA. Looking at the results in Table 3, with a higher level of PES, the mediated effect tends to decrease gradually, while the predictive effect of IU on SA becomes stronger. This is because PES is stable and habitual. Once a high PES is formed, it is easier for individuals to develop a negative explanation of fuzzy information than to ruminate about the meaning of that fuzzy information (Gonzalez-Diez et al., 2017). This result is in accordance with the theory of differential activation (Teasdale, 1988). It suggests that to improve college students’ rumination of SA about fuzzy information, we should assess their personal tolerance to uncertain situations and distinguish their explanatory style types. According to the theory of acquired helplessness (Seligman et al., 1968), PES is not innate; rather, it is constantly learned in the acquired environment. Encouraging college students to explain life events positively and optimistically and reduce PES formation in daily life may effectively reduce the possibility of SA.

The moderated mediation model proposed in this study reveals the moderating effects of PES on the first part of the mediation process and the direct path from IU to SA. The results indicate that a high or low pessimistic interpretation style will negatively affect an individual’s cognition. When counseling an individual with SA, the focus should be on decreasing anxiety, but it is also important to consider their explanatory style and rumination types. Considered in previous research, it is feasible to improve college students’ tolerance of uncertainty by improving the attachment type (Yildiz and Iskender, 2019). Based on our research results, in the process of counseling and providing interventions for college students with SA, it seems to be able to pay more attention to change their pessimistic explanatory about ambiguity. For example, when we use exposure-therapy in counseling, it can be more effectively when based on evaluations of ambiguity than focusing on negative social events. Also, cognitive reconstruction or rational counseling techniques can be used to alter the explanatory style of an individual with SA. In addition, we can reduce rumination through mindfulness therapy (Bishop et al., 2004), which will help to alleviate the adverse effects of SA on college students.

There are some shortcomings in this study. Firstly, the data were self-reported by the subjects. It is universally acknowledged that the results could be influenced by social desirability and other factors. Future studies can use multiple methods to improve the reliability and validity of studies. In addition, the relationship between IU and SA can be affected by other cognitive factors that were not considered in present study. Therefore, more cognitive factors should be included in our further research, such as: self-focused attention, post-event processing, and catastrophic misinterpretation (Morrison and Heimberg, 2013; Luo et al., 2018). Secondly, the cross-sectional design makes it is difficult to assess the long-term validity of the results and it also constrain us from drawing conclusions about causality. Future studies can involve an intervention group and a control group as well as a longitudinal design. Finally, this study only investigated the SA of Chinese college students, which limits the generalizability of our study findings. Based on cultural limitations of psychological research (Ye, 2004), whether college students in different cultural environments show similar results deserves further research. Recent studies found that Chinese college students’ social anxiety scores are significantly higher than American college students’ norm scores (Zhang et al., 2020). Future research can replicate our model by comparing the SA of different cultural groups of college students using transnational samples.

Despite some limitations of the study, the results provide valuable information regarding the issue of SA in college students in China. Given the negative impact of SA on college students, more research is needed to better understand the mechanisms by which risk factors contribute to SA. Our study provides both a theoretical and an empirical basis for the development of interventions by exploring the mediated mechanism. The model shows the interaction of cognitive factors that induce SA, which supports several classic models of social anxiety and also provides a reference for extending the relationship between IU and SA. In particular, it provides unique ideas for counseling and treatment of social anxiety college students from the perspective of changing the negative explanatory style.

## Conclusion

The main conclusions of this study are as follows:

(1) IU was positively correlated with PES, rumination, and SA. There was a significant positive correlation between rumination and SA.
(2) Rumination plays a partial mediating role in the relationship between IU and SA.
(3) The association between IU and SA and the mediating effect of rumination are moderated by PES. The higher the PES level, the stronger the relationship between IU and SA, and the weaker the mediating effect of rumination.

## Data Availability Statement

The raw data supporting the conclusions of this article will be made available by the authors, without undue reservation.

## Ethics Statement

The studies involving human participants were reviewed and approved by the Moral & Ethics Committee of School of Psychology, Jiangxi Normal University (Nanchang, China). The patients/participants provided their written informed consent to participate in this study.

## Author Contributions

JL conceived the idea of the study, performed the survey, and approved the final version of the manuscript to be published. XC and YX analyzed the data and contributed to the writing of the manuscript. YX, JL, and SL contributed to the revised manuscript. All authors contributed to the article and approved the submitted version.

## Conflict of Interest

The authors declare that the research was conducted in the absence of any commercial or financial relationships that could be construed as a potential conflict of interest.
